# Unusual presentation of intramuscular hemangioma in abdominal oblique muscles

**DOI:** 10.1002/ccr3.5383

**Published:** 2022-04-04

**Authors:** Jameel Miro

**Affiliations:** ^1^ Surgical Oncology Umm Al‐Qura University Mecca Saudi Arabia; ^2^ Surgical Oncology Doctor Soliman Fakeeh Hospital Jeddah Saudi Arabia

**Keywords:** hemangioma, oblique muscles, outcome

## Abstract

Intramuscular hemangiomas are rare benign tumors that are difficult to diagnose. We report a successful case of intramuscular hemangioma excision involving the external oblique muscles. The mass was excised successfully, and histopathology confirmed the diagnosis of intramuscular hemangioma with a negative margin and no malignancy.

## Graphical abstract

Intramuscular hemangiomas are rare benign tumors that are difficult to diagnose. We report a patient with a 3‐year history of a mass that was excised successfully. Histopathology confirmed the diagnosis of an intramuscular hemangioma.

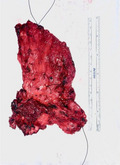

## 1 INTRODUCTION

Intramuscular hemangiomas (IMHs) were first reported by Liston in 1843,^1^ and Allen and Enzinger subsequently conducted the first large‐scale study of 89 patients in 1972.^2^

Vascular malformations are usually benign, rare tumors, accounting for less than 1% of all hemangiomas.^3^, ^4^ The most common sites are the limbs, followed by the head and neck. The abdominal wall muscle is the rarest site for intramuscular hemangiomas. Generally, hemangiomas are slow‐growing masses.^5^ However, they can also mimic malignant tumors.^3^

More than half of patients with intramuscular hemangiomas report pain (55%)^4^ and swelling, with persisting symptoms from 1 to 5 (range, 0–70) years.^6^ Hemangiomas may have purpuric discoloration and superficial dilated veins, typically from its cutaneous extensions. In nearly all cases (98%), a mass is found that can be pulsatile or have a bruit.^7^ The mass is usually movable in the transverse direction but not along the line of the fibers. Because of its rarity and vague presentation, more than 90% of IMHs are misdiagnosed before surgery.^8^ Therefore, an imaging modality is essential, and the modality of choice for defining the vascular nature of the tumor^8^ and providing soft tissue delineation and spatial involvement of the lesion is magnetic resonance imaging (MRI), which is better than computed tomography (CT).^9^

In 1972, Allen and Enzinger ^2^ suggested a classification based on the size of the predominant vessel type involved, corresponding to the type of hemangioma (small vessel, <140 mm in diameter is capillary; large vessel, 140 mm in diameter is cavernous and mixed type). Tumor histopathology reveals blood vessels in addition to various amounts of fibrofatty tissue, smooth muscle, thrombus, and bone, with minimal differences between types.^2^, ^6^

There have been limited studies on anterior abdominal wall muscle hemangiomas.^3^, ^10^, ^11^, ^12^, ^13^, ^14^, ^15^ We present a case of abdominal wall hemangioma involving the external oblique muscle. To the best of our knowledge, this is the first case reported in the Middle East.

## 2 METHODS

Patient data were gathered prospectively from hospital electronic records. A literature review was performed using PubMed and Google Scholar search engines using the keywords “hemangioma,” “abdominal hemangioma,” “hemangioma and rectus,” and “intramuscular hemangioma.” No statistical analysis was required for this case report.

## 3 CASE REPORT

### 3.1 Clinical history/examination

The patient was a 21‐year‐old man with no known medical illness other than a torn external adductor muscle in his left thigh 2 years previously that was treated conservatively. The patient had no surgical history. He presented to the general surgery clinic with a history of a mass that was neither growing nor painful and had been present for the past 3 years. Examination showed a soft, compressible, nonpulsatile mass with indistinct borders on the right flank.

### 3.2 Differential diagnosis, investigation, and management

**FIGURE 1:**
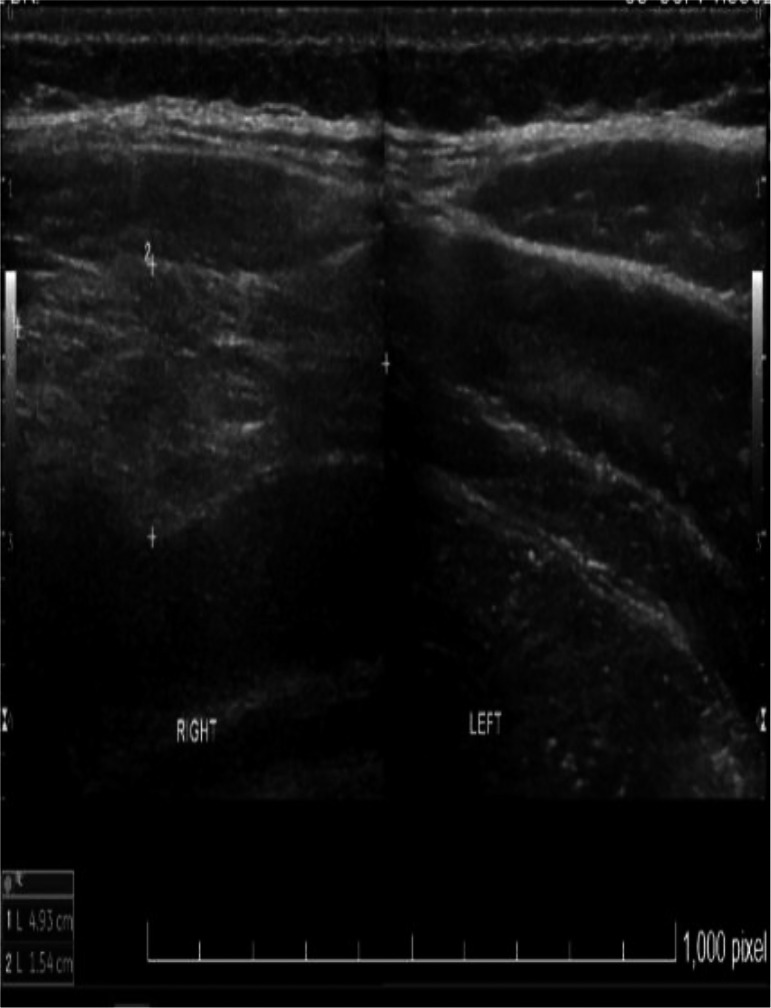
Ultrasound of soft tissue showing a large echogenic solid lesion in the anterior abdominal wall on the right side between the abdominal wall muscles measuring approximately 10 × 8 × 2.4 cm

Ultrasound (US) of the soft tissue (Figure 1) revealed a large echogenic solid lesion measuring approximately 10 × 8 × 2 cm in the anterior abdominal wall on the right side between the abdominal wall muscles. This finding was likely an intermuscular lipoma requiring further MRI evaluation.

**FIGURE 2:**
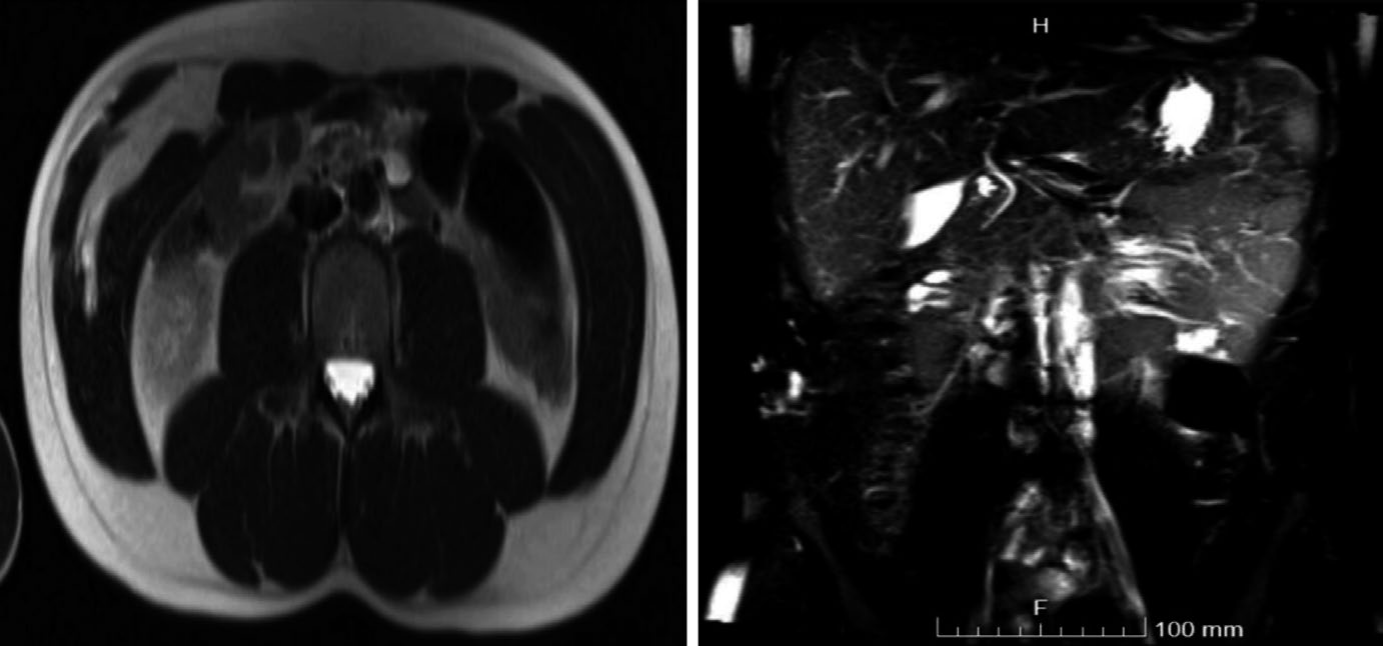
Magnetic resonance imaging of the abdomen with contrast (axial and coronal) showing a large intramuscular mass in the right anterior abdominal wall, extending from the lower edge of the costal margin to a few centimeters above the inguinal ligament (15 cm)

Abdominal MRI with contrast (Figure 2) showed a large intramuscular mass in the right anterior abdominal wall measuring approximately 8.3 × 9 × 1.9 cm composed predominantly of adipose tissue. The presence of internal linear and nodular high T2 components that showed minimal diffusion restriction and enhancement was highly suspicious for well‐differentiated liposarcomas rather than lipomas. Another possibility was the presence of a hibernoma.

**FIGURE 3:**
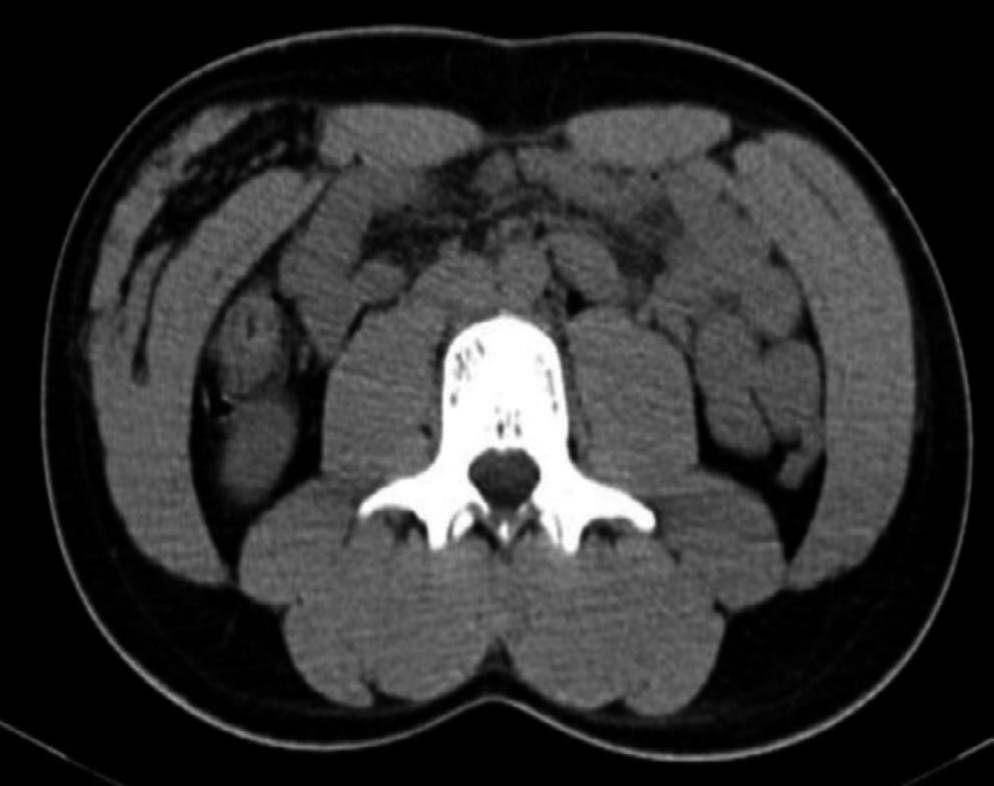
Computed tomography‐guided biopsy revealing histopathology consistent with intramuscular hemangioma

A CT‐guided biopsy (Figure 3) suggested intramuscular hemangioma, and no malignancy was observed in the submitted tissue.

The patient was informed of the results, and consent for surgery was obtained with an explanation of the risks and benefits of surgery as well as alternative treatments. It was explained that in case of muscle invasion and/or features of neoplastic lesions, a compartmental excision of the abdominal wall with reconstruction with alloplastic material might be needed.

### 3.3 Surgery

The patient was placed in a supine position under general anesthesia. After preparing the abdomen and draping in the usual manner, we observed a lateral bulge on the right side of the abdominal wall, which the surgeon marked preoperatively. MRI showed that the mass extended from the lower edge of the costal margin to a few centimeters above the inguinal ligament (15 cm). The mass lay immediately below the external oblique muscle and aponeurosis. We decided to perform an oblique flank incision because the medial extension was only to the medial border of the rectus muscle.

A 10‐cm incision was made. The fascia of Scarpa was incised and lateralized with skin elevation. The external oblique muscle and aponeurosis were then incised in the direction of the fibers. Minor bleeding was encountered in the highly vascularized tumor below the muscle. The external oblique was mobilized medially until the rectus muscle was observed. The rectus muscle was not invaded, and the mass was easily dissected.

A ligature bipolar device was used for dissection and coagulation. The entire medial border of the tumor was easily removed. The caudal side was thin (spike‐like) and could be exposed and mobilized. The lateral side was somewhat difficult to distinguish and dissect from the internal oblique superficial fibers. We decided to remove some of the superficial fibers and dissect them anatomically. The upper border was freed from the lower edge of the costal margin.

**FIGURE 4:**
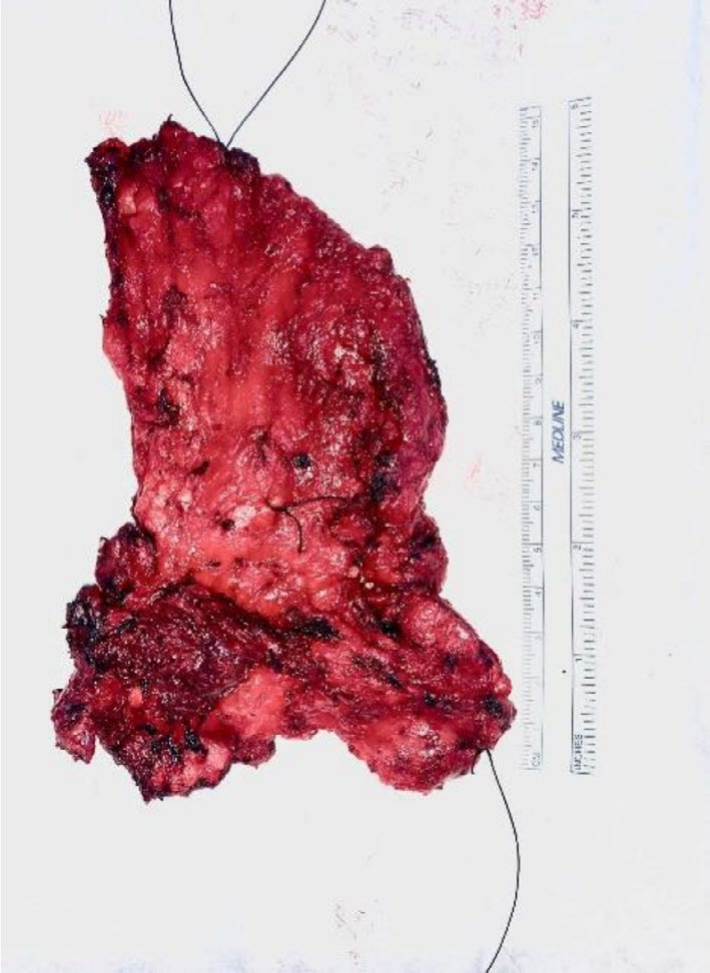
Excised external oblique muscle and hemangioma tissue

The specimen was retrieved and marked for histopathological examination (two long superior, two short anterior, and one long inferior; Figure 4). Hemostasis was achieved using bipolar forceps and irrigation of the surgical site. A 15‐Fr drain was inserted, and closure of the external oblique was achieved with running 0 Vicryl suture and the fascia of Scarpa with 2–0 Vicryl suture. Monocryl sutures were used for skin closure. Infiltration of the wound and drain site with local anesthesia was performed, and a dressing was applied.

### 3.4 Outcome and follow‐up

The patient recovered well, and the drain was removed 4 days postoperatively. Abdominal wall mass excision result was consistent with intramuscular hemangioma.

The patient was examined in the general surgery clinic 2 weeks postoperatively. He was doing well with no pain, and the wound healed. The patient underwent abdominal and pelvic US follow‐up after 1 year.

## 4 DISCUSSION

Vascular malformations are usually benign, rare tumors accounting for less than 1% of all hemangiomas.^3^, ^4^ They are slow‐growing masses,^5^ but because they mimic malignancy,^3^ they need to be properly investigated.

There have been limited studies on anterior abdominal wall muscle hemangiomas with only five cases involving the abdominal rectus muscles reported,^3^, ^10^, ^11^, ^12^ one case involving the internal oblique muscle,^1^ and two cases involving the lateral abdominal wall (unspecified).^13^, ^14^ Interestingly, another study mentioned a hemangioma that involved three abdominal wall layers.^15^ Our case was intramuscular hemangioma of the external oblique muscle.

Clinically, hemangiomas are misdiagnosed in nearly all cases (90%).^5^ Therefore, imaging is required. Diagnostic imaging with Doppler imaging, MRI, angiography, and Tc‐99m erythrocyte localization provides remarkable results.^3^, ^4^, ^16^, ^17^

In our study, US of the mass showed a large echogenic solid lesion in the anterior abdominal wall on the right side. This was followed by MRI with contrast showing a large intramuscular mass in the right anterior abdominal wall composed predominantly of adipose tissue with internal linear and nodular high T2 components showing minimal diffusion restriction and enhancement, which are highly suspicious for well‐differentiated liposarcomas rather than lipomas. Therefore, CT‐guided biopsy was recommended to confirm the diagnosis of intramuscular hemangioma.

Spontaneous resolution is unusual and may cause local destruction over time owing to the effect of pressure on nearby structures.^10^, ^18^, ^19^ Thus, surgical resection is warranted to remove the lesion, relieve pain, and exclude malignancy. An adequate surgical margin is important to avoid local recurrence, but it can be difficult to achieve for deep infiltrating intramuscular hemangiomas.^20^ In our case, the patient underwent wide excision of the mass, and histopathological examination was performed to exclude local recurrence and the risk of malignancy. Fortunately, the tumor was completely excised with no malignancy.

## 5 CONCLUSION

Intramuscular hemangioma is difficult to diagnose and its presentation is frequently unusual. We demonstrated that proper planning and imaging modalities are needed to successfully diagnose and manage intramuscular hemangiomas. Wide excision of the mass is required to decrease the risk of malignancy.

## CONFLICT OF INTEREST

No conflict of interest.

## AUTHOR CONTRIBUTIONS

The author confirms sole responsibility for the following: study conception and design, data collection, analysis and interpretation of results, and manuscript preparation.

## CONSENT

Written consent has been obtained from the patient.

## Data Availability

The data supporting the findings of this study are available from the corresponding author upon reasonable request.
